# Role of galectin-glycan circuits in reproduction: from healthy pregnancy to preterm birth (PTB)

**DOI:** 10.1007/s00281-020-00801-4

**Published:** 2020-06-29

**Authors:** Sandra M. Blois, Stefan Verlohren, Gang Wu, Gary Clark, Anne Dell, Stuart M. Haslam, Gabriela Barrientos

**Affiliations:** 1 Experimental and Clinical Research Center, A Cooperation Between the Max Delbrück Center for Molecular Medicine in the Helmholtz Association and the Charité–Universitätsmedizin Berlin, AG GlycoImmunology, Berlin, Germany; 2 Institute for Medical Immunology, Charité-Universitätsmedizin Berlin, Berlin, Germany; 3 Department of Obstetrics and Fetal Medicine, University Medical Center Hamburg-Eppendorf, Hamburg, Germany; 4 Department of Obstetrics, Charité-Universitätsmedizin Berlin, Berlin, Germany; 5 Department of Life Sciences, Imperial College London, London, UK; 6 Department of Obstetrics, Gynaecology and Women’s Health, University of Missouri, Columbia, Missouri USA; 7 Laboratory of Experimental Medicine, Hospital Alemán, School of Medicine, University of Buenos Aires, CONICET, Buenos Aires, Argentina

**Keywords:** Galectins, Preterm birth, Microbial infections, Glycans

## Abstract

Growing evidence suggests that galectins, an evolutionarily conserved family of glycan-binding proteins, fulfill key roles in pregnancy including blastocyst implantation, maternal-fetal immune tolerance, placental development, and maternal vascular expansion, thereby establishing a healthy environment for the growing fetus. In this review, we comprehensively present the function of galectins in shaping cellular circuits that characterize a healthy pregnancy. We describe the current understanding of galectins in term and preterm labor and discuss how the galectin-glycan circuits contribute to key immunological pathways sustaining maternal tolerance and preventing microbial infections. A deeper understanding of the glycoimmune pathways regulating early events in preterm birth could offer the broader translational potential for the treatment of this devastating syndrome.

## Introduction

Galectins play a paramount role in pregnancy biology, modulating a wide range of processes from embryo implantation to parturition. Different galectins coexist at the feto-maternal interface where besides coordinating placentation and maternal immune adaptation to the semi-allogenic fetus, they also play a role in maternal vascular expansion [1]. Though most of their biological functions during gestation are exerted through binding endogenous glycan structures, galectins can also recognize exogenous specific glycans on the surface of bacteria, viruses, parasites and therefore function as pattern recognition receptors [2]. As a result, galectins appear to be critical in the microbial glycan-host interactions that promote the engagement of specific immune cell subsets and shape host immunity. Thus, given their unique ability to modulate maternal immunity galectins emerge as important players in preterm birth (PTB) syndrome, which most often is associated with microbial infections that disrupt fetomaternal tolerance due to the drastic link between underlying pathogens and their ability to promote inflammatory responses [3, 4].

Host-pathogen interactions fundamentally shape a broad range of biological processes. While products of microbial metabolism can impact a wide variety of host activities, from neurological function to overall metabolism and immune homeostasis (1–3), direct interactions between host and microbes can fundamentally shape microbial flora, impact immune function and often ultimately dictate the likelihood of infectious disease (4). Although host factors can interact with a variety of distinct microbial molecular determinants, cell surface glycans represent the most unique, diverse, and rich molecular features that decorate microbes (5, 6). As microbial carbohydrate determinants often completely envelope microbes, these structures often represent the first and most significant molecular signature encountered by a host. As a result, hosts appear to have evolved a variety of immune factors that possess the ability to recognize the distinct carbohydrate signature of a broad range of microrganisms (6–9). Indeed, many immune populations are defined by the distinct repertoire of glycan-binding proteins (GBPs) they express (6–8), strongly suggesting that microbial glycan-host interactions may result in the engagement of specific immune cells and thus shape host immunity in fundamental ways.

### Galectins and the control of pregnancy-associated processes

**Fig. 1:**
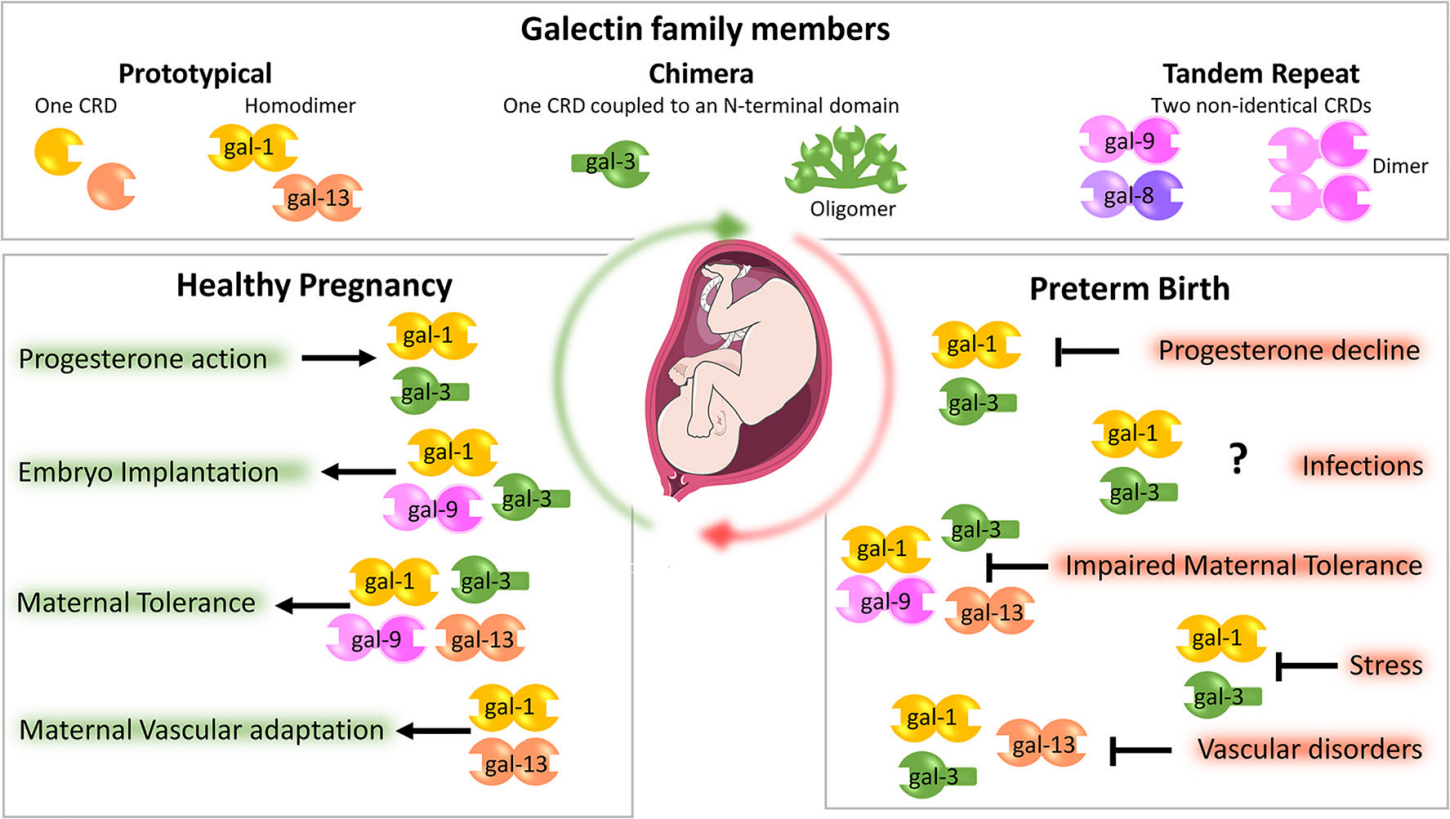
Biological function of galectins at the feto-maternal interface. The galectin family members are divided into three types: the prototype with one carbohydrate recognition domain (CRD), the chimeric type with one CRD and a non-lectin N-terminal domain and the tandem –repeat type with two CRDs connected by a non-conserved linker. Some galectins can self-associate into dimers or oligomers. Under normal conditions, individual galectins promote healthy gestation regulating placentation, maternal immune and vascular adaptation to pregnancy. Progesterone induces the expression of galectin-1 (gal-1) and gal-3 during embryo uterine receptivity. Factors implicated in the development of preterm birth are likely to contribute locally to galectin dysregulation and as a consequence breakdown of maternal immune tolerance and vascular disorders may trigger spontaneous preterm labor

Galectins are small, soluble glycan-binding proteins characterized by their affinity to β-galactosides and the presence of an evolutionarily conserved sequence, the carbohydrate recognition domain (CRD), which mediates binding to their specific N-acetyllactosamine [Galβ(1–4)-GlcNAc]-enriched ligands [5]. In mammals, 15 members of the galectin family have been identified so far, of which 13 are expressed in humans [6]. Based on their molecular structure, they are classified into three main types: prototype, chimera, and tandem-repeat galectins (Fig. 1). While some of these galectins contain one CRD and are biologically active as monomers (i.e., gal-1, gal-13) or as oligomers that aggregate though their non-lectin domain (gal-3); others contain two CRDs connected by a short linker peptide (e.g., gal-9). Galectins are synthesized in the cytoplasm, where they exert intracellular functions modulating various processes including cell growth, differentiation, survival, and migration [7]. In addition, some galectins can translocate to the nucleus and participate in transcriptional regulation and mRNA splicing [7, 8]. However, galectins can also be present on the cell surface or secreted to the extracellular compartment [9], where they engage in protein-glycan interactions with cell surface or ECM molecules and regulate a diverse combination of biological functions such as cell adhesion, apoptosis, lattice formation, and invasion [10–13]. With their various functions, galectins link innate and adaptive immune responses acting as key regulators of acute and chronic inflammation, host-pathogen interactions, and immune tolerance, which all are implicated in a healthy pregnancy [14–18].

### Embryo implantation

It is now firmly established that the role played by galectins during the establishment and maintenance of gestation is attributable to the several pathways these endogenous lectins coordinate. Figure 1 illustrates the galectin-associated mechanisms during a healthy gestation. Evidence from in vitro and expression studies suggest galectins are important mediators in the implantation process. Indeed, during embryo implantation the increased expression of gal-1, -3, and -9 in endometrial epithelial cells suggest their role in uterine receptivity [19–22]. In support of this, the ability of galectins to bind laminin and fibronectin [23] may serve as a link between endometrial epithelial cells and the blastocyst. Human embryos express gal-1 at early stages of development (day 3–day 5) in their trophectoderm and secrete gal-1 into the medium in which they are cultured [24], suggesting that this lectin may influence uterine blastocyst attachment during the window of implantation. In this regard, Jeschke’s group has shown that gal-1 binds mucin-1 (MUC1) via the Thomsen-Friedenreich (TF) epitope on glandular epithelial cells and endometrial epithelial apical surface tissue [25], implying that embryonic-derived gal-1 may bind to endometrial MUC1 via the TF epitope during implantation. Interestingly, integrins (e.g., αβ3) have been proposed to have important roles during implantation [26] and the integrin β3, which is highly expressed in the luminal and glandular epithelium, could also serve as a ligand of gal-1 and gal-3 to promote trophectoderm-uterine epithelium interactions [27]. Thus, galectins (especially gal-1, gal-3, and gal-9) participate not only in the uterine epithelial preparation for receptivity but also in blastocyst activation influencing the embryo-derived signals for implantation.

### Maternal Tolerance

The establishment and maintenance of pregnancy represent a major immunological challenge requiring a delicate balance of inflammation and immune tolerance at the fetal-maternal interface. During early stages, proper implantation and uterine vascular adaptation are characterized by an inflammatory milieu, which later must be switched to a down-modulation of the immune response allowing tolerance of the semi-allogenic fetus. Later on, a new switch to inflammation is required in the last stage to ensure the activation of labor. This key immune switching mechanism at the fetal–maternal interface relies on a highly orchestrated crosstalk involving the placental trophoblasts and different maternal immune cell subsets, such as regulatory macrophages, natural killer (NK) cells, and T cells (recently reviewed in [28]). Thus, maternal immune adaptation to pregnancy is a highly regulated process involving several galectins [6, 15, 29–31]. In this regard, pioneering studies by Than NG et al. have shown that placenta-specific galectins (e.g., gal-13, -14 and -16), predominantly expressed by the syncytiotrophoblast cells, induce maternal T lymphocyte apoptosis [32]. In a more recent work, Than’s group showed that gal-13 and gal-14 have a basic pro-apoptotic activity on T cells regardless of their activation status [33]. However, cytotoxic T lymphocytes were more susceptible to gal-13/gal-14 induced apoptosis than T helper cells, probably due to the differential glycosylation pattern on these two T cell populations [34]. In addition, gal-1 is highly expressed in the hemochorial placenta, where it has been shown to modulate human leukocyte antigen G (HLA-G) expression on extravillous trophoblast (EVT) cells, thereby promoting one of the chief mechanisms of immune tolerance operating at the human maternal–fetal interface [24]. The immune regulatory effects of HLA-G include impacts on NK cell killing activity, suppression of cytotoxic T lymphocyte killing activity and viability, inhibition of proliferation and induction of a suppressive phenotype in T helper cells, and alteration of dendritic cell maturation and stimulatory capacity (reviewed in [35]). The expression of gal-9 by human trophoblast has been shown to promote the development of uNK cells with a tolerogenic phenotype via Tim-3 engagement [36], which is supported by data indicating that the Tim-3/gal-9 pathway downregulates Th1 immunity [37]. Additionally, galectins are also expressed by maternal immune cells, which infiltrate the decidua. For example, gal-1, secreted by uterine natural killer (uNK) cells, induces the apoptosis of activated decidual T cells with a glycophenotype compatible with this lectin [38]. uNK cells also selectively express type 2 β-1,6-*N*-acetylglucosaminyl transferase (C2GNT), the glycosylation enzyme required to initiate the formation of gal-1 specific ligands, implying an autocrine role of this lectin in down-modulating the cytotoxic potential of uNK cells [38]. Gal-9 has an immunosuppressive activity similar to gal-1 at the maternal side [39]. The effect of Lgals9 *D5* (the predominant gal-9 splice variant) was tested on uNK cells in mice and it was found to downregulate IFN-gamma production through carbohydrate dependent interaction [39]. Thus, gal-9 could participate in the limitation of Th1 and shift to a protective Th2 milieu, which is further supported by the impaired decidual expression of gal-9 in mice and human pregnancy complicated with spontaneous abortion induced by T helper cytokine imbalances [39]. The ability of gal-1 to maintain the balance between pro-inflammatory Th1/Th17 and Th2 cytokines needed for healthy gestation is critical. We have shown that gal-1 promotes the expansion of IL-10 producing regulatory T cells [15]. In line with these findings, *LGALS1* null mice display exacerbated Th1/Th17 responses and a higher frequency of immunogenic DC [34, 40] and show increased fetal loss rates in allogeneic pregnancies with susceptibility to stress-induced abortions [15, 41]. In summary, these evidences support a role for galectins in dampening inflammatory responses and promoting tolerogenic cell phenotypes specifically at the fetal–maternal interface. During pregnancy, this serves as a mechanism of promoting maternal tolerance to the fetus through preventing deleterious anti-fetal T cell responses.

### Maternal vascular adaptations for placental development

A proper placental development requires a deep maternal vascular adaptation in early gestation. In this regard, different steps of the angiogenic cascades and endothelial cell biology are influenced by galectins (e.g., gal-1, gal-3, gal-8, gal-9) [42]. For instance, several lines of evidence demonstrate proangiogenic functions for gal-1, which result from direct effects on endothelial cell activation via H-Ras signaling [43] as well as from the modulation of endothelial cell adhesion, migration and proliferation by interacting with the neuropilin (NRP)-1/VEGFR2 signaling pathway [44]. Murine studies have demonstrated a critical role of VEGFR2 signaling during the physiological adaptation of the maternal vascular bed to embryo implantation [45], which together with the high local expression of NRP-1 during peri-implantation stages [46] points out to a paramount role played by this lectin in the control of pregnancy angiogenic responses. Indeed, treatment with anginex (an artificial β-peptide targeting gal-1 proangiogenic functions) resulted in decreased adhesion and capillary tube formation in SGHPL-4 EVT-like cells in vitro and impaired spiral artery remodeling and placental function in an in vivo mouse model, causing preeclampsia-like symptoms during late gestation and fetal growth restriction [47].

Another galectin likely to be involved in maternal vascular adaptation is gal-13 (placental protein 13, PP13), though evidence in support of its role per se in the modulation of angiogenic pathways is still elusive. In decidual tissue, gal-13 is found selectively associated with T-cell-, neutrophil-, and macrophage-rich foci of necrosis [48], suggesting that it might act to attract, activate and kill maternal immune cells facilitating trophoblast invasion and spiral artery remodeling. More recently, in vivo studies demonstrated hypotensive effects in pregnant rats infused with gal-13 [49], associated with increased heart rate and decreased peripheral resistance due to general vasodilation. It was later demonstrated that gal-13 infusion both during pregnancy and in the non-pregnant state was associated with vasodilation of veins and resistance arteries beyond the uterine vascular tree [50, 51], suggesting that placenta-derived gal-13 may be involved in generating a systemic endothelial effect in the mother mediated by endothelial nitric oxide synthase (eNOS) and prostaglandin signaling.

### Galectins in parturition

Parturition is a coordinated process referred to as the “common pathway” that involves increased myometrial contractibility, cervical ripening, activation of the decidua, and fetal membranes with local pro-inflammatory changes. These processes involve different uterine compartments including the decidua, myometrium, fetal membranes, and placenta implying that activation of biological pathways may be different across the various gestational tissues. As galectins are widely expressed, we intend to discuss the galectin signature of gestational tissues at term taking into consideration the origin of the expression. However, due to the intimal interaction between decidua, chorion, and amnion, it may be difficult to infer the galectin expression pattern of these tissues separately. Although data is relatively scarce, evidence suggests that at term, gal-1 is the galectin with the highest expression in the human decidua. In healthy laboring women, gal-1 and gal-3 expression levels within the decidua decreased when compared to non-laboring women [52]. Consistent with this, maternal gal-9 circulating levels are elevated early in healthy pregnancy and remain increased until parturition, returning to non-pregnant levels in the post-partum period [53]. In pregnant mice, gal-3 is mainly expressed in the endometrial cells of the primary decidua basalis, metrial gland, and placenta; and after parturition this lectin expression decreased as the implantations sites resorbed [54], implying that the parturition process at least in humans and mice occurs with a dysregulation of the glycan-binding proteins.

### PTB, a complex syndrome associated with multiple causes

**Fig. 2:**
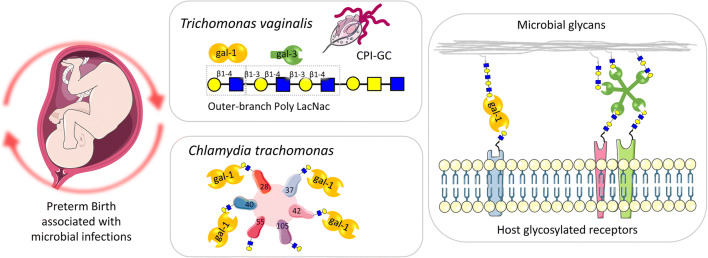
Microbial-induced inflammation and galectin-glycan circuits. Schematic diagram to illustrate galectin functions as pattern-recognition receptor for microbes associated with preterm labor. Galectin-1 (gal-1) and gal-3 specifically bind the N-glycans displayed in the Lipophosphoglycan (LPG). Gal-1 is able to bind at least six chlamydia trichomonas glycoproteins (gp28, gp37, gp40, gp42, gp55, and gp105). Galectins may facilitate the ascendant infection during pregnancy by cross-linking host and microbial glycans. Abbreviations: Ceramide phosphoinositol glycan core (CPI-GC); gp glycoprotein

PTB is defined as birth before 37 + 0 gestation weeks (GW). However, the simplicity of the definitions is in sharp contrast to the complexity of the disease, whose etiology is far from being understood. PTB is the leading cause of neonatal morbidity and mortality and the single major cause of death in children up to 5 years of age in the developed world [55]. About 15 million preterm neonates are born each year where genetic variation in human birth timing imposed a high risk for prematurity in the African American population [56]. In the European Union, the PTB rate has risen constantly over the last 10 years, a trend which corresponds to global figures [57, 58]. The prevalence varies from country to country with a median of 7.1% of all births. In Germany, 9% of all children were born before the end of GW 37. In parallel, the rate of extremely preterm deliveries (< 28 GW) has risen by 64%. Neonates that are born preterm are at an increased risk of short-term and long-term complications, with the former being attributed to the immaturity of multiple organ systems and the later ranging from disabilities originating from these early complications to subtle neurodevelopmental impairment [59] (Fig. 2).

Although preterm labor has a complex, multifactorial etiology [60] microarray data from uterine tissue revealed similar gene regulation patterns between term and preterm women suggesting that acceleration of the gestational clock appears to be involved in the PTB cases. In particular, spontaneous preterm labor is perceived as pathological activation of the above mentioned “common pathway” of parturition. Other key factors for labor, cervical ripening, and decidual/membrane activation, involve specific changes in inflammatory and extracellular matrix proteins. These include increased expression of inflammatory cytokines e.g. tumor necrosis factor-α (TNF-α) and IL-1 and chemokines, increased activity of proteases matrix metalloprotease 8 (MMP-8) and MMP-9, degradation of extracellular matrix components such as fibronectin and an increase in glycosaminoglycans and hyaluronan [61]. In addition, pro-inflammatory pathways including chemokines (interleukin-8 (IL-8)), cytokines (IL-1 and -6), and contraction-associated proteins (oxytocin receptor, connexin 43, prostaglandin receptors) eventually contribute also to myometrial activation [62].

While many studies examined the roles of galectin interactions during gestation, the potential outcome of these interactions in the context of PTB remains elusive. Shankar and co-workers [63] have identified differences in choriodecidual gal-1 expression between spontaneous preterm labor and gestational-matched non-laboring patients, suggesting that decreased levels of gal-1 are associated with the underlying pathology. Early studies demonstrated that galectins are expressed in cervical and vaginal epithelial cells [64], which could uniquely poise to engage microbes and initiate innate immunity. Indeed, the observation that gal-1 is able to downregulate the pro-inflammatory environment stimulated by LPS (e.g., IL-6 production, an important cytokine related to PTB) in decidual cells derived from elective cesarean patients at term suggests that this lectin may be important in the regulation of local inflammation during the course of chorioamnionitis [65]. Similarly, it has been shown that gal-3 is increased in fetal membranes and in the amniotic epithelium in patients with chorioamniotic infection [66], thereby regulating the inflammatory response and/ or direct interaction with the pathogens. In the following sections, we discuss several of the multiple pathological processes associated with preterm labor and the relevance of galectin-induced immune-regulatory pathways:

### Decline in progesterone and anti-inflammatory mediators

Progesterone is a key player in maintaining uterine quiescence, and a withdrawal of this hormone is observed at parturition onset. An understanding of this phenomenon has led to the successful application of progesterone in threatened preterm labor [67]. Moreover, a recent study has shown that progesterone treatment could serve as an anti-inflammatory strategy to prevent PTB and adverse neonatal outcomes induced by T cell activation [68]. While current data suggest that progesterone regulates endometrial galectin expression including gal-1 and gal-3 [69] and alterations of the progesterone receptor function during gestation associate with reduced levels of gal-1 expression [15, 70], future studies will likely determine whether alterations in galectin expression directly contribute to PTB pathophysiology.

### Microbial infection

Microorganism-induced PTB is mediated by an inflammatory process and the most studied mechanism is the activation of toll-like receptors (TLRs). TLRs are membrane-bound proteins that recognize pathogen-associated molecular patterns (PAMPs) and activate the innate immune system to generate downstream signals through the release of cytokines (IL-1β, TNF-α), chemokines (IL-8, CCL-2), prostaglandins and proteases [71]. The activation of the innate immune response through TLRs has the aim to control microorganisms that may injure the embryo, however, excessive inflammation could eventually trigger the common pathway of parturition with cervical ripening, rupture of fetal membranes and placental detachment [61, 72]. TLRs can be expressed in the cell surface (TLR-1, -2, -4, and -5) or in intracellular vesicles (TLR-3, -7, -8, and -9). Cell-surface TLRs recognize accessible PAMPs such as bacterial lipoproteins and lipoteichoic acid (TLR-2 as heterodimers with TLR-1 or TLR-6), lipopolysaccharide (LPS) of Gram-negative bacteria (TLR-4) or bacterial flagellin (TLR-5). Cytoplasmic TLRs recognize double-stranded RNA (dsRNA) (TLR-3), single-stranded RNA (ssRNA) (TLR-7 and TLR-8) or CpG enriched double-stranded DNA (TLR-9) [71].

In human pregnancies, TLR-1 to TLR-10 have been found and are mainly expressed by trophoblast cells, but also in the cervix and uterus [73, 74]. However, differential expression of TLRs has been observed according to the gestational age. Particularly, in the third-trimester placenta, the expression of TLR-2 was observed in endothelial cells, macrophages, and syncytiotrophoblast and TLR-4 was prominently expressed in syncytiotrophoblast and endothelial cells [75]. Functional analysis also demonstrated that the term placenta can respond to TLR-3, TLR-5, and TLR-7/8 agonists [76]. In a mouse model, it has been demonstrated that activation of TLR-3 with poly(I:C), an analog of dsRNA, promotes NF-kappa B signaling with the induction of pro-inflammatory cytokines and chemokines (e.g., IL-6, IL-1β, TNF-α, IFN-γ, IL-8, MCP-1), leading to preterm delivery [77]. Moreover, in women with chorioamnionitis TLR-2 and TLR-4 are upregulated in fetal membranes [78]. Administration of peptidoglycan, which is part of the bacterial cell wall, induced TLR-1 and TLR-2-mediated trophoblast cell death in vivo and in vitro. However, apoptosis could be inhibited by the presence of TLR-6, which also activates NF-kappa B signaling in trophoblasts with the secretion of IL-6 and IL-8 promoting an inflammatory response [79]. Both the pro-inflammatory response and trophoblast apoptosis are processes strongly implicated in PTB. As TLR-4 recognizes LPS it is not surprising that TLR4-deficient mice are not susceptible to LPS- or *Escherichia coli*–induced PTB [80, 81] and neutralizing antibody against TLR-4 can reduce inflammation-induced PTB and fetal death in mice [82]. In rhesus monkeys, pretreatment with a TLR-4 antagonist inhibited LPS-induced uterine contractility and reduced IL-8, TNF-α, and prostaglandins [83]. Treatment with IL-10 prevented LPS-induced PTB with a reduction of TNF-α, IL-6, and IL-1β in mice and rats [84, 85].

From the clinical point of view, associations between microbial induced inflammation and preterm labor have been reported in several studies [86–88], but it is not clear why some women experience PTB and some not, even with the same exposure to pathogens. A striking example is a discrepancy between the rate of lower genital tract and ascending intra-amniotic infections, implicating that the role of the maternal immune system is key to identify those at risk. Nevertheless, in 25% of all PTBs intra-amniotic infection is involved [89]. Ascending infections are the likely cause, as pathogens detected in the amniotic fluid and in the lower genital tract are the same [90]. Recently, the PREMEVA trial investigated the effect of screening and therapy for bacterial vaginosis (imbalance of naturally occurring bacterial flora with an increase of the anaerobic type) in pregnant women with a low or high risk of preterm labor (according to previous PTB history) treated or not with clindamycin, which is one of the two most often-used antibiotics to treat bacterial vaginosis during pregnancy. The authors concluded that bacterial vaginosis treatment in women with low-risk pregnancies did not show a reduction of spontaneous PTB suggesting that the use of antibiotics to prevent preterm delivery should be reconsidered [91]. This finding is in line with other previous randomized controlled trials and meta-analyses that have shown no effect of antibiotics for pregnancy prolongation in asymptomatic pregnant women with bacterial vaginosis [92–94]. In addition, recent studies report that positive diagnosis with *Chlamydia trachomatis* (the most common aerobic intracellular bacterium responsible for sexually transmitted infections) shows no significant association with spontaneous preterm labor [95]. However, other trials have shown that women with chlamydia infections are 2.28 more likely to deliver pre-term in comparison with those who were not infected [96]. Group B *Streptococcus* (GBS, a gram-positive bacterium) colonization is recognized as a risk factor for PTB as being the most frequent cause of severe early-onset infection in newborn babies. A recent review showed a consistent increase in the risk of PTB in women with maternal GBS colonization, which is stronger in case-control studies compared to cohort or cross-sectional studies [97]. For *Trichomonas vaginalis*, a vaginotropic extracellular protozoan parasite, similar results have been retrieved. Women with asymptomatic trichomoniasis were randomly treated with metronidazole or placebo. Preterm delivery occurred in 19% of the metronidazole vs 10.7% in the placebo group. While metronidazole eliminated the organism, it was reported as ineffective in preventing preterm delivery and potentially even increasing it, which has led to early termination of the trial [98].

In the context of microbe recognition, galectins can directly engage microbes by binding specific glycans on their surface and thereby dictate the consequence of microbial exposure [99]. Thus, galectins can function as both pattern recognition receptors (PRRs) and innate immune effectors during microbial infections [100], promoting pathogen clearance through different mechanisms (i.e., phagocytosis, encapsulation, autophagy) or inhibiting adhesion and/or entry into the host cell. This recognition and effector role can however be ‘subverted’ by certain pathogens, which can take advantage of the host galectin repertoire for successful attachment, invasion, and immune evasion [100, 101]. An example of this strategy relevant for PTB is the *Trichomonas vaginalis* lipophosphoglycan (LPG) and its immunocompetent ceramide phosphoinositol glycan core (CPI-GC) domain-containing β-galactosides and abundant poly-N-acetyl-lactosamine repeats [102], which provide targets for gal-1 and gal-3 recognition [64]. Recent studies in this area of research have demonstrated that gal-1 mediates the adherence of the parasite to cervical epithelial cells in an LPG-dependent manner [103]. Moreover, CPI-GC collected from multiple clinical isolates showed similar affinity to gal-1, but the affinity to gal-3 differed between isolates from different patients, suggesting that galectin-binding diversity may responsible for the *Trichomonas* symptom disparity [64]. Another valid example of preterm labor infection is *Chlamydia trachomatis*, with gal-1 being able to bind to at least six chlamydial glycoproteins (gp28, gp37, gp40, gp42, gp55, and gp105). Thus, gal-1 facilitates *C. trachomatis* infection by bridging bacterial and host glycosylated receptors (such as PDGFRβ and β_1_/α_V_β_3_ integrins) [104]. As intrauterine ascension of pathogens through the vaginal tract is one of the routes of pathogenic entry that triggers preterm labor, all aspects regarding recognition properties of the female tract (vagina, cervix, and placenta) galectin repertoire and the dynamic of their subcellular compartmentalization/secretion and interactions with microbial carbohydrates warrant further investigations.

Evidence indicates a possible association between galectins and TLRs. A well-studied example in the context of infection-induced PTB is group B streptococci, bacteria that produce membrane vesicles with extracellular matrix-degrading proteases and pore-forming toxins leading to collagen degradation in the chorio-decidual membranes [105]. Interestingly, in vitro stimulation of cord blood samples with an invasive strain of *Streptococcus agalactiae* (a group B *Streptococcus*) induces gal-3 expression. Since fetal gal-3 serum levels increase with gestational age, the authors speculated that impaired gal-3 expression may contribute in part to the high susceptibility of preterm infants to infection as opposed to term infants or adults [106]. In addition, RNA interference against TLR-3 was shown to prevent gal-9 expression in human umbilical vein endothelial cells (HUVECs) stimulated with poly(I:C) [107]. In line with this finding, activation of TLR-3 (by poly(I:C)) and TLR-4 (by LPS) on fibroblasts derived from rheumatoid arthritis patients lead to apoptosis protection through induction of gal-9 expression [108]. Moreover, gal-9 was increased upon stimulation with poly(I:C) in hepatitis C virus-infected monocytes [109]. With these evidences, we could hypothesize that activation of TLR-3 (principally by dsRNA produced by the virus) could increase gal-9 exerting a pro-inflammatory effect. It has also been described that gal-3 knockdown human synovial fibroblasts stimulated with an agonist to TLR-2 (Pam3CSK4), TLR-3 (poly(I:C)-), or TLR-4 (LPS) display a reduced response to TLR-mediated IL-6 secretion, suggesting gal-3 functions as a positive regulator of TLR activation [110].

Some evidence indicates a possible association between periodontal infection caused by oral pathogenic bacteria (e.g., *Campylobacter rectus* (*C. rectus*) or *Porphyromonas gingivalis* (*P. gingivalis*)) and PTB [111]. Enhanced placental TLR-4 expression was observed after oral infection with *C. rectus* and *P. gingivalis* [112]. In a mouse model with *P. gingivalis* infection, TLR-2-induced inflammation in the fetal membrane (activation of NF-kappaB and p38 MAPK pathways) leads to the upregulation of uterine contractility causing preterm delivery [113]. Similarly, *P. gingivalis* LPS induced IL-6 and IL-8 production via TLR-2 in human chorion-derived cells [114]. Interestingly, increased gal-3 was found in the placenta, amniotic fluid, and serum in a PTB model of *P. gingivalis*–infected mice. In vitro culture of HTR-8/SVneo trophoblast cells with *P. gingivalis* LPS, demonstrated increased levels of TNF-α and gal-3, and gal-3 inhibition significantly downregulated *P. gingivalis* LPS-induced TNF-α production [115]. During the neuroinflammatory response, it was demonstrated that gal-3 associates with TLR-4 through its CRD [116]. Moreover, *P. gingivalis* LPS increased gal-9 expression in the human periodontal ligament (connective tissue fibers) [117] suggesting a role for gal-9 during infection-induced PTB.

We have demonstrated that stress challenge during early pregnancy can enhance permeability of mucosal membranes to the entry of bacterial products (e.g., LPS) and promote transmucosal migration of commensal bacteria inducing fetal loss in mice [41]. Stress-triggered fetal loss was prevented by blocking of TLR-4 (anti-TLR-4 antibody) or neutralization of LPS (using the bactericidal/permeability-increasing protein (BPI), a protein that specifically binds and neutralizes LPS). In addition, gal-1 deficient female mice were highly prone to stress-triggered complete implantation failure, but treatment with BPI markedly reduced the detrimental effect of stress in pregnancy outcomes. However, there are not data available regarding the susceptibility to ascending infections and PTB in gal-1 deficient mice. The anticipated role of gal-1 as a key factor against pathogen mediated PTB suggests that insufficient gal-1 could be a critical factor that predisposes some women to infection-mediated PTB.

### Clinical management

Screening for PTB consists in determining risk factors by taking a detailed history of the pregnant woman. Ideally, potential risk factors such as status post (s/p) previous PTB, short inter-pregnancy interval et al are determined before pregnancy allowing for preventive strategies. General primary prevention includes cessation of smoking and treatment of bacterial vaginosis in pregnancy [118]. However, in women with a history of PTB, the prophylactic treatment with vaginal progesterone or even a prophylactic cerclage may be considered [119]. General screening for PTB such as routine measurement of the uterine cervix by transvaginal sonography is not recommended. However, sonographic assessment of the cervical length should be included in the diagnostic work-up in symptomatic pregnant women (regular spontaneous preterm contractions) and/or in women with risk factors for spontaneous PTB [120]. In addition to transvaginal sonography, biomarkers such as PAMG-1, fetal fibronectin, and phIGFBP-1 obtained from cervico-vaginal secretions may be used to specify the risk of a PTB within the next seven days [121]. In women with a sonographic short cervix, secondary prevention consists of treatment with vaginal progesterone [122]. Treatment with a cervical pessary has not demonstrated to decrease the rate of spontaneous early preterm delivery [123]. Before 24 weeks, cervical cerclage may be the treatment of choice [124]. It should be noted that these measures have only proven to be effective in singletons and not in multifetal gestations.

The main aim of tertiary prevention in the context of threatened PTB before 34 weeks of gestation is to prolong pregnancy for at least 48 h in order to allow for the antenatal corticoid application. There is broad international consensus that placenta-crossing steroids (betamethasone, dexamethasone) must be given to women at imminent risk for PTB before 34 weeks in order to accelerate organ maturation of the fetus [125]. In order to achieve that, tocolysis, emergency cerclage, progesterone, and vaginal pessary can be used, adapted to the clinical situation and after counseling, ideally involving a multidisciplinary team including a neonatologist. Accurate assessment of the remaining pregnancy duration is paramount in order to find the best timing of steroid application as the ideal window is seven days before birth. Preterm premature rupture of membranes (PPROM) requires balancing the risks between prolongation of pregnancy for maturation and timely delivery in order to prevent the potentially devastating complications of ascending intrauterine infections.

In our view, management of threatened PTB is largely symptom-driven and preventative and the therapeutic strategies are guided not by causative approaches, but rather by preventative measures. Research in the field of PTB must be intensified in order to clarify the underlying etiologies allowing for targeted strategies in the future. Insights into the galectin-glycan circuits of tissues such as myometrial smooth muscle cells, decidua, placenta, amnion as well as fetal and maternal blood are sparse [62]. Identifying specific glycoimmune phenotypes, as well as factors capable of modulating maternal immune responses, can help to better predict which women might be at risk for preterm labor, permitting better surveillance and prophylaxis.

### Galectin-glycan circuits as modulators of inflammation and infection: insights from pregnancy

Glycans are essential functional groups that facilitate and influence the reproduction process. For instance, the embryo implantation process is driven by glyco-specific interactions between the uterine epithelium and the outer trophoblast cell layer of the blastocyst, such that perturbations of the system generally result in implantation failure or poor pregnancy outcomes. Glycosylation relies on a delicate balance in the activity of specific modification enzymes (glycosyltransferases and glycosidases), and the glycocode expressed in a particular tissue is highly dependent on the cell type and its developmental, nutritional and pathological state. The specific glycome expressed at the maternal–fetal interface can play multiple roles during pregnancy. For example, N-linked glycans (attached to the nitrogen of an asparagine side-chain) have been shown to modulate trophoblast invasion [126, 127] and maternal–fetal tolerance [128, 129] during placentation. O-Linked glycans (attached to the hydroxyl oxygen of serine, threonine, tyrosine, hydroxylysine, or hydroxyproline side-chains) can influence recognition events during fertilization (e.g., sperm-egg interactions) [130].

**Fig. 3:**
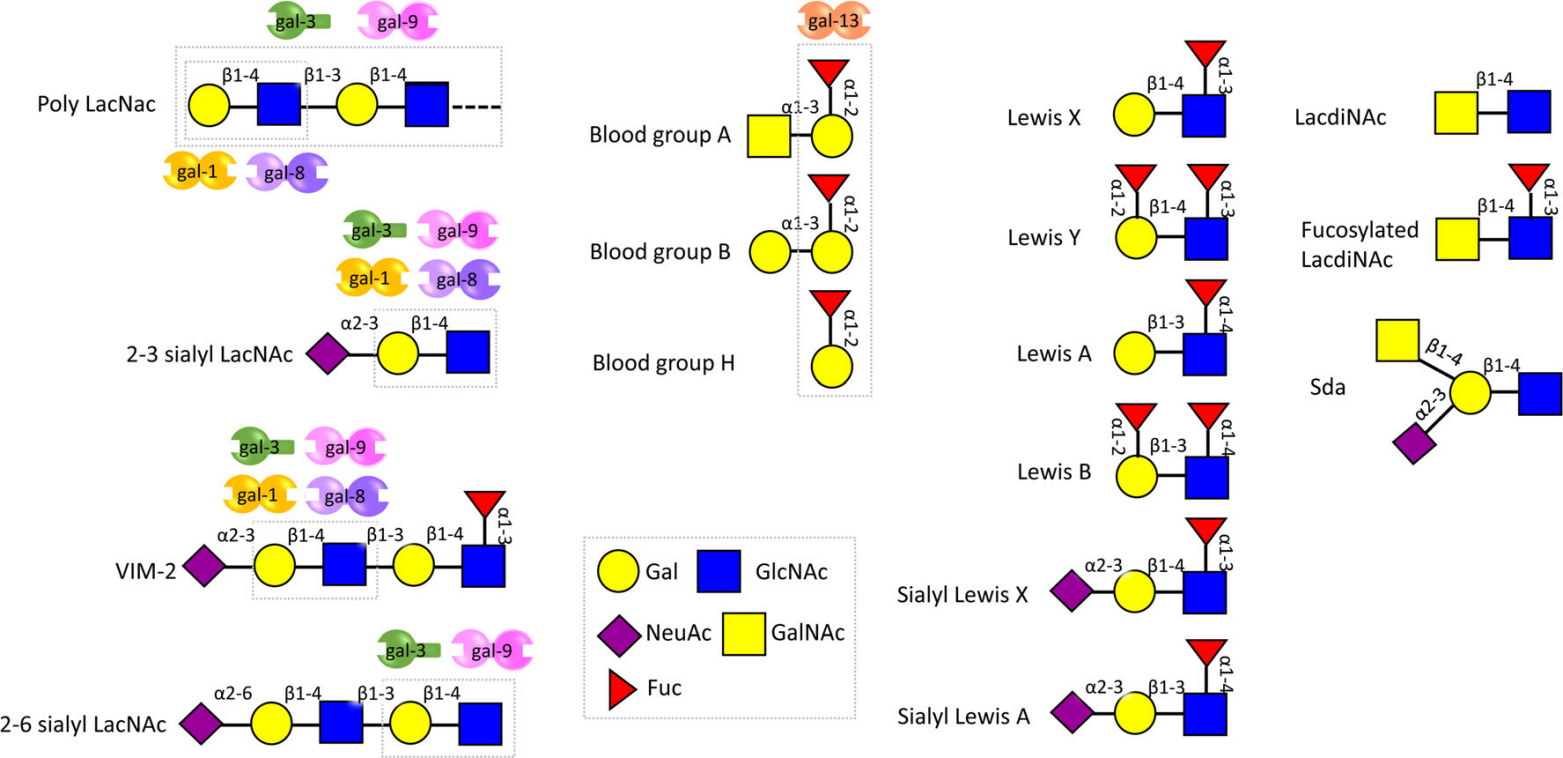
Glycan terminal structures found in the reproductive system. The LacNac structures can be potential ligands for galectins expressed in the reproductive system, and the binding epitopes are shown within rectangles. Modifications of LacNac structures by sialic acid and fucose could either block or enhance galectin binding. Abbreviations used in the figure: gal galactose; GlcNAc N- acetylglucosamine; NeuAc Neuraminic Acid; Fuc Fucose

Extracellular functions of galectins depend on the cross-linking of surface N- and O-glycans expressed by maternal immune cells, trophoblasts, and endothelial cells at the fetal-maternal interface (Fig. 3). As glycosylation is directly related to the physiological cellular status, changes in glycan composition are highly regulated during pregnancy and can have a fundamental impact on galectin activity [131–133]. For instance, placental expression of N-acetylglucosaminyl transferase V (GnTV), which generates the β1-6-N-acetylglucosamine branches in complex N-glycans recognized by gal-1, is enhanced in the first trimester compared with term pregnancies [126]. Since gal-1 promotes EVT differentiation and invasion during early pregnancy [134], it is possible that increased activity of GnTV may lead to enhanced signaling by this lectin [127]; particularly by promoting its interaction with cell surface β1 integrin [127, 135–141] Furthermore, villous tissues from early spontaneous miscarriages show a reduced abundance of such (β-6) branches together with decreased GnTV expression in comparison with healthy pregnancy villous tissues [142]. Thus, differences in the glycan composition of trophoblast related-proteins at the same gestational age could be important disease biomarkers that await further investigation. Indeed, the placental expression of GnTV was reported to be elevated in preeclampsia compared to normal pregnancies [143]. Thus, increased gal-1 expression as we have demonstrated in late-onset preeclampsia could represent a protective mechanism of the trophoblast to overcome the severe inflammatory milieu that characterizes the syndrome [47]. This is an interesting example of how the metabolic status of trophoblast cells is reflected by their glycan signature, which is shaped by the intracellular levels of GnTV expression affecting the quality and branching of complex N-glycans and therefore modulating galectin binding.

### Glycosylation in pregnancy, term, and preterm labor

Protein glycosylation is of fundamental importance at all stages of human pregnancy from conception and implantation to delivery. Especially important are antennae sequences of N- and O-glycans which are potential ligands for lectins. Figure 3 displays the repertoire of glycan epitopes which are known to be expressed in the human male and female reproductive tracts. Both human gametes express functionally important glycoproteins. Glycoproteins of the extracellular matrix zona pellucida of the oocyte, present the sialyl-LexisX sequence [NeuAcα2-3Galβ1-4(Fucα1-3)GlcNAc] as their major terminal structures on both N- and O-linked glycans. This terminal sialyl-LexisX sequence was shown to be the ligand that mediated sperm-egg binding [144].

Characterization of human sperm N-glycans identified high mannose, biantennary complex glycans with bisecting GlcNAc and multi-antennary complex N-glycans with LewisX and LewisY [Fucα1-2Galβ1-4(Fucα1-3)GlcNAc] sequences. Such N-glycan structural features have been associated with inhibition of both the adaptive and innate immune systems [145]. High levels of LewisX and LewisY sequences were also observed on human seminal plasma N-glycans (and O-glycans), but levels of biantennary complex glycans with bisecting GlcNAc were reduced. This altered glycosylation profile would be more implicated with inhibition of the adaptive immune system and less so with inhibition of the innate immune response [146]. The presence of immunosuppressive glycans on sperm and in seminal plasma might function in improving fertilization outcomes [147].

Some of the most studied pregnancy-associated cell types are trophoblast cells, which are required for the formation and maintenance of the placenta and therefore functionally mediate the exchange of gases, transport of nutrients, and hormone production. There are different sub-types of trophoblast cells, EVT, which invade decidua and spiral arteries and are important in maintaining maternal blood supply into the placenta, syncytiotrophoblasts (STB), which are located on the villous surface and therefore are the primary cellular interface between the maternal blood supply and placental villi, and cytotrophoblasts (CTB), which are located just below the STBs. Glycomic characterization of the N-glycans of these cells revealed both common and differential glycan structural features. All three cell types expressed abundant high mannose glycans and dominance of α2-3 linked sialylated glycans over α2-6 linked. All cell types also expressed complex biantennary glycans with bisecting GlcNAc, but levels in EVT were lower than in CTB and STB. In contrast, EVT expressed higher levels of multiantennary and polylactosamine extended complex N-glycans compared to CTB and STB. The α2-3 linked sialylated termini and the polylactosamine antennae are both potential ligands for galectins.

A genome-wide association study (GWAS) has found that slit guidance ligand 2–roundabout guidance receptor 1 (SLIT2-ROBO1) signaling in trophoblasts is associated with PTB and that higher mRNA levels of *SLIT2* and *ROBO1* are detected in the basal plate of placentas from PTB samples [148]. Interestingly, knockdown of *ROBO1* in trophoblast-derived cells upregulated 6 of 10 pregnancy-specific glycoproteins (PSGs). PSGs are members of the immunoglobulin superfamily and are produced by trophoblast cells and pass into the maternal blood supply during pregnancy. All 10 PSGs have been implicated in immunomodulatory functions [149] and are important for the maintenance of normal pregnancy [150]. PSGs contain multiple potential N-glycosylation sequences and also potential sites for O-glycosylation and evidence of glycosylation have been indicated by lectin binding studies [151]. Recently, we have characterized PSG1 n detail in terms of its glycosylation [152]. We showed that PSG1 contains multi-antennary complex N-glycans with high levels of α2-3 sialic acid capping. Low levels of N-glycans with bisecting GlcNAc were also observed. In addition, we demonstrated that PSG1 specifically interacts with gal-1-1 with an estimated K_D_ of 0.13uM. Of potential functional importance, the binding of PSG1 by gal-1 protected it from oxidative inactivation.

The best-characterized pregnancy-associated glycoprotein in amniotic fluid is glycodelin A (GdA), which is a member of the lipocalin family of proteins. Lipocalins are a large family of small proteins that share tertiary structures and, except for glycodelin, typically transport or store small biological compounds such as vitamins and steroid hormones. Schiefner et al. [153] have determined the crystal structure of GdA and showed that it forms a dimer that presents its N-glycans in an array format conducive to high-affinity lectin binding. Interestingly the glycodelin gene has only been found in humans and higher primates and Schiefner et al. have pointed out that the occurrence of glycodelin coincides with the evolution of menstruation in higher primates. The detailed structural characterization of GdA N-glycosylation showed that two of the three potential N-glycosylation sites are occupied and that there is site-specific glycosylation. Asn-28 carries high mannose, hybrid, and complex-type structures whereas Asn-63 exclusively carries complex glycans [154]. Subsequent analyses using more sensitive mass spectrometry methodologies revealed the presence of a more complex glycome including tri- and tetra- antennary complex structures carrying the Sda epitope (NeuAcα2–3(GalNAcβ1–4)Gal)[155]. Interestingly, it has also been demonstrated that GdA from women with gestational diabetes mellitus (GDM) have altered N-glycan structures with reduced levels of α2-6 sialylation and high mannose glycans and an increase in levels of Sda epitopes. These glycosylation changes correlated with reduced immunosuppressive activity in in vitro assays [156]. It has recently been reported that the odds of PTB are 30% higher in women with GDM [157].

T cells are a well-defined target of GdA. GdA has been discovered to inhibit T cell proliferation in response to allogeneic antigens [158] and induce apoptosis of activated T cells [159]. Chronic chorioamnionitis (CCA) is the process of amniotropic infiltration of maternal T cells, which can break maternal/fetal tolerance and lead to maternal anti-fetal allograft rejection [160]. CCA is one of the major placental lesions of spontaneous preterm birth and is considered as the most common pathology of late preterm birth [161]. Proteomic analysis of amniotic fluid samples has found that GdA is significantly lower in CCA, compared to the samples from acute chorioamnionitis and gestational age-matched controls [162]. These results suggest a pathophysiological link between preterm birth and GdA. The glycans on GdA may be associated with maternal tolerance to fetal antigens and PTB.

Human chorionic gonadotropin (hCG) is another essential pregnancy-associated glycoprotein. Recent glycomic studies have shown that hCG from pregnant women corresponded to mono-, bi-, tri-, and tetra-antennary N-glycans. There was also a substantial amount of bisected N- glycan structures with abundant LewisX capping. Interestingly hCG from women later diagnosed with pre-eclampsia also showed a high abundance of sialylated bi-antennary N-glycans [163].

Dynamic changes in cervical glycosaminoglycans (GAGs) have been found during pregnancy [164]. Six types of GAGs have been identified: hyaluronan (HA), dermatan sulfate (DS), keratan sulfate (KS), chondroitin sulfate (CS), heparin, and heparan sulfate (HS). However, HA exclusively increases from 19% in early pregnancy to 71% at term. In addition, the size of HA decreases in labor, due to higher activity of HA digesting enzyme hyaluronidase. The changes of HA during pregnancy are hypothesized to contribute to cervical ripening for term and PTB.

A potential cause of PTB are infections ascending from the vagina to the intrauterine cavity through the cervical tube [4]. The change of permeability of the cervical tube is related to preterm birth [165]. Mucin glycoproteins are a major constituent of mucus along the cervical tube, which functions as a physical barrier against ascending bacteria. These proteins are extensively O-glycosylated and the O-glycans are heavily clustered in Ser/Thr rich domains, which are separated by short non-glycosylated regions. These O-glycan chains can be terminated by ABO blood groups, Lewis antigens, and sialic acid. As glycans can be used as receptors by many bacterial adhesins during infection, the change of cervical mucus glycans could alter the microbiome in the intrauterine cavity.

## Concluding remarks and future perspectives

Current evidences have established that galectins have multiple roles in healthy gestation and regulate the immune response during infections. However, our understating of the role of galectins in parturition is scarce and key priorities to further reveal their contribution include (1) defining the galectin signature during healthy parturition and preterm labor, (2) delineation of the mechanism (e.g., glycan structures) by which galectins regulate ascending infections and orchestrate the immune response against microbes, and (3) identifying galectins as possible regulators of cervical remodeling and uterine senescence that may predispose to cervical dysfunction and preterm labor in women. Focused studies in animal models and human tissue are likely to reveal the galectin-glycans circuits over the onset of labor and post-partum tissue repair.
